# Structural characterization of a novel polysaccharide from *Sargassum thunbergii* and its antioxidant and anti-inflammation effects

**DOI:** 10.1371/journal.pone.0223198

**Published:** 2019-10-04

**Authors:** Dianhui Luo, Zhaojing Wang, Kaiying Nie

**Affiliations:** Department of Bioengineering and Biotechnology, Fujian Provincial Key Laboratory of Biochemical Technology, Huaqiao University, Xiamen, Fujian, People’s Republic of China; Institute of Medical Research and Medicinal Plant Studies, CAMEROON

## Abstract

A novel polysaccharide STSP-I was isolated and purified from *Sargassum thunbergii*. Its structure and bioactivity were studied using gas chromatography (GC), fourier transform infrared spectroscopy (FTIR), periodate oxidation-smith degradation, partial acid hydrolysis, methylation-GC-MS, nuclear magnetic resonance (NMR), transmission electron microscopy (TEM), radicals scavenging assays and anti-inflammatory assays. STSP-I was consisted of fucose and galactose with a molar ratio of 1.2:1, and its mass was 373 kDa. The main structural components of STSP-I were →4)-α-D-Gal*p*-(1→ and →3)-β-L-Fuc*p*-(1→, STSP-I was a non-branched polysaccharide, and TEM further revealed the existence of entangled chains and linear forms. Compared with Vitamin C (Vc), STSP-I showed a higher scavenging effect of superoxide radical (EC_50_ = 0.22 mg/mL) and an equivalent scavenging effect of hydroxyl radical (EC_50_ = 0.88 mg/mL). STSP-I also exhibited good inhibitory effects of TNF-α, IL-6 and COX-2 mRNA expressions in LPS-stimulated RAW 264.7 mouse macrophage cells, and the inhibitory effects were more than 91% at the concentrations of 75 and 150 μg/ml. The results indicate that the polysaccharide STSP-I from *S*. *thunbergii* with the linear structure may serve as potential antioxidant and anti-inflammatory agents.

## Introduction

Marine algae are considered to be the rich sources of biological active compounds [1]. *Sargassum thunbergii*, as one of brown algae, has been mainly used for abalone bait [2]. It comprises abundant compounds, such as isopentadiene, phlorotannins, polysaccharides and proteins [3–4].

Polysaccharides from *S*. *thunbergii* have attracted increasing attention due to its biological and pharmacological functions [5–7]. Fu et al [8] reported that 5-linked arabinose, 3-linked mannose, 3,6-linked galactose, 6-linked glucose and 3-linked xylose are the main linkage types of a heteropolysaccharide prepared from *S*. *thunbergii*. Jin et al [9,10] proved that ST-1 might contain many backbones by ESI-MS spectra, including sulfated galacto-fucan, sulfated glucuronomannan or fucoglucuronan, and ST-2 contained sulfated galacto-fucans branched with sulfated fuco-oligomers. Zhuang et al [11] indicated that the polysaccharides consisted mainly of fucoidan or L-fucan on the basis of chemical and spectral analyses. We studied structural properties of a major polysaccharide (STP-II) prepared from *S*. *thunbergii* [12–13].

Inflammation occurs through physical stimulation, such as infections and tissue injuries, and macrophage plays key roles in inducing inflammatory reactions by releasing different types of cytokines [14–16]. Macrophage activation are derived from gram-negative bacteria with lipopolysaccharides (LPS), and several inflammatory factors including tumor necrosis factor (TNF-α), interleukin (IL-6) and cyclooxygenase (COX-2) can be released to protect bodies [17]. However, over-expression of the inflammatory factors is involved in a number of diseases, such as atherosclerosis, chronic obstructive pulmonary disease, rheumatoid arthritis and autoimmune diabetes [18–20]. Thus, inhibition of inflammatory factors is believed to be crucial for managing these diseases, and many investigators have been concentrated on researching anti-inflammatory compounds from natural resources [21–23].

Here in our study, we obtained a novel polysaccharide which isolated from *S*. *thunbergii* using the alkaline-extraction, we examined its structure and testified its anti-inflammatory effect, and the results will be benefit for the further investigation of *S*. *thunbergii* in medical and food industries.

## Materials and methods

### Materials and chemicals

*Sargassum thunbergii* was purchased from a local store (Dalian, Liaoning Province, China), then dried at room temperature, the brown algal was identified by Professor J. F. Liu, Huaqiao University, Fujian, China. Monosaccharide and dextran standard samples, 3-(4,5-dimethylthiazol-2-yl)-2,5-diphenyltetrazolium bromide (MTT), phenazine methosulfate (PMS), lipopolysaccharide (LPS) and dimethyl sulfoxide (DMSO) were obtained from Sigma Chemical Co. (St. Louis, MO). DMEM medium (Hyclone, Logan, UT), fetal bovine serum (FBS, Hyclone, Logan, UT), Penicillin-Streptomycin (MP Biomedicals, CA, USA), and RAW 264.7 mouse macrophage cells (American type culture collection, ATCC) were purchased from a local agent (Taijing Co., Xiamen). Ascorbic acid (vitamin C, Vc), hydrogen peroxide (H_2_O_2_), Nitro Blue Tetrazolium (NBT), Nicotinamide Adenine Dinucleotide Hydrogen (NADH) and ferrous sulfate (FeSO_4_) were obtained from Shanghai Macklin Chemical Reagent Company, China. DEAE Sepharose CL-6B was obtained from Pharmacia, Co, now available from GE-Healthcare. Eastep Total RNA Super Extraction Kit (LS1040), GoScript Reverse Transcription System (A5001) and GoTaq qPCR Master Mix (A6001) were purchased from Promega Co. (Madison, WI, US). All reagents were of analytical grade.

### Extraction and purification of polysaccharides

Dry *S*. *thunbergii* powder was extracted using the hot-water extraction method [13], the residues were dried at room temperature, and the dry *S*. *thunbergii* was extracted again by adding 0.5 mol/L of NaOH solution at 4°C for 10 h, adjusting pH = 2.0 with 1 mol/mL of glacial acetic acid and adding salivary amylase at 37°C for 2 h. The extraction solution was concentrated and added four volumes of ethanol at 4°C for 24 h, the precipitations were dried to produce ST, and protein was further removed by using Sevage method and freeze-thawing cycles to give STSP. STSP (500 mg) was dissolved in 10 mL of distilled water, the supernatant was injected into a column (4.6 cm×40 cm) of DEAE-Sepharose CL-6B, and the column was eluted with distilled water at 50 mL/h (12 min/tube), followed by eluting with NaCl aqueous solution (0 to 1 mol/L). The carbohydrate fraction corresponding to a major peak was collected from the water aqueous solution and dialyzed against tap water and distilled water for 48 h. The dialyzed solution was concentrated and dried in a freeze drier to produce a purified polysaccharide STSP-I.

### Homogeneity, molecular weight (MW) and physicochemical properties

High-performance size-exclusion chromatography (HPSEC) was used to analyze the homogeneity and molecular weight (MW) of STSP-I as described previously [24–25], and 1100 system (Agilent Technologies, Palo Alto, CA, USA) equipped with a Shodex Sugar KS-804 column (Showa Denko K.K, Japan) was used for the research.

Total sugar content was determined using the phenol-sulfuric acid method [26], the concentration of proteins was measured according to Bradford’s method [27], and sulfate content was determined by Wang’s method [28].

The identification and quantification of monosaccharide composition of STSP-I were identified by gas chromatography (GC). STSP-I (30 mg) was hydrolyzed with 1 mol/L of H_2_SO_4_ at 100°C for 8 h, neutralized with barium carbonate, and freeze-dried to obtain hydrolysis products. The derivatization of hydrolysis products was carried out with a trimethyl chlorosilane (TMCS) reagent [29], and the derivative products were analyzed by using a gas chromatography system (6890 system, Agilent Technologies, Palo Alto, CA, USA) fitted with an HP-5 column (30 m×0.25 mm×0.25 μm) and a flame-ionization detector (FID).

For FTIR measurement, STSP-I (3 mg) was ground with 100mg of KBr powder, pressed into pellets, and putted in a Fourier Transform Infrared Spectrometer (Nicolet iS10, Thermo Fisher Scientific, Waltham, MA, USA) with a DTGS detector.

### Structural characterization

For the partial hydrolysis with acid, STSP-I (100 mg) was hydrolyzed with 0.05 mol/L of CF_3_COOH and dialyzed, the product outside the bag filter (MWCO 3500) was used for GC analysis, and the precipitate and the supernatant in the bag filter were for GC analysis [30].

Periodate oxidation-Smith degradation were used for the analysis of glycosidic linkages. STSP-I (25 mg) was oxidized and reduced, and the oxidized-reduced polysaccharides were hydrolyzed with 1 M sulfuric acid for Smith degradation as described previously [30]. 1/3 neutralizing solution was freeze-died for GC analysis (S1), and the remaining neutralizing solution was dialyzed. The dialysis solution (S2) was freeze-died for GC analysis, the solution inside the dialysis tube was added 4 volumes of ethanol, and the supernatant (S3) and the sediment (S4) were used for GC analysis.

The methylation of STSP-I (30 mg) was carried out with methyl iodide, and the methylated polysaccharides were extracted, reduced and acetylated as described previously [30]. The resulting methylated alditol acetates were subjected to a HP7890A-5975C instrument (Agilent Technologies, Palo Alto, CA, USA) with an HP-5ms (30 m×0.25 mm×0.25 μm) column for gas chromatography mass spectrometry (GC-MS) analysis.

For nuclear magnetic resonance (NMR) measurements, STSP-I (30 mg) was dissolved in 0.7 mL of D_2_O (99.96%), and acetone was added into the solution as the internal standard. NMR experiments were performed on an AVANCE-500 NMR spectrometer (Bruker Inc., Rheinstetten, Germany) at 27°C, including ^1^H, _13_C, ^1^H/^1^H homonuclear correlation spectroscopy (^1^H-^1^H COSY), total correlation spectroscopy (TOCSY), heteronuclear single quantum coherence (HSQC), and heteronuclear multiple-bond coherence (HMBC) [31–33].

### Microscopic detection

The polysaccharide STSP-I with sodium dodecyl sulfate (SDS) was observed under the transmission electron microscopy (H-7650, Hitachi High-Technologies Corporation, Tokyo, Japan) [30].

### Antioxidant activities detection

The superoxide, hydroxyl and DPPH radical scavenging activities were measured using the methods previously described [12, 24]. The percentage of the radical scavenging activity was calculated according to the following equation.

$$Scavenging\;activity\;(\%)\;=\;[1\;-\;(A_{0}/A_{1})]\;\times \;100\%$$

In the equation, A_0_ was the absorbance of the blank control, and A_1_ was the absorbance of the sample.

### Cytotoxicity assay

The cytotoxicity of STSP-I against RAW264.7 cells was determined using MTT assay. RAW264.7 cells were cultured in DMEM medium (high glucose) containing 10% of FBS and 100 units/mL of penicillin-streptomycin with 5% of CO_2_ at 37 °C, and they were seeded in 96-well culture dishes at a dose of 100 μl/well for 20 h after growing 80% of confluent. DMEM medium was discarded, and 100 μl of medium, containing different concentrations of STSP-I (75, 150, 300, 600 and 1200 μg/mL), were added to 96-well culture dishes for 24h. After that, the cytotoxicity was determined by adding 20 mL of MTT (5 mg/mL) to each well and incubating for 4 h, removing the media, adding 150 μL of DMSO to each well and measuring absorbance at 570 nm. The inhibition rate was calculated from the following equation.

$$Inhibition\;rate(\%)=[1-(A_{570nm,sample}/A_{570nm,control}]\;\times \;100\%$$

### Anti-inflammatory measurements

According to the above result, STSP-I was dissolved into DMEM medium at two concentrations of 75 and 150 μg/mL, and LPS (1μg/mL) was used as the negative control. The potential anti-inflammatory effect of STSP-I was investigated by discarding DMEM medium after culturing RAW 264.7 cells in 6-well plates to reach an 80% confluence, adding 2 mL of STSP-I medium for 24 h, and adding LPS at a final concentration of 103BCg/mL for 4 h. After that, the culture medium was discarded, and the cells were collected for total RNA isolation [34].

According to the manufacturer’s instructions, total RNA was isolated using Eastep Super total RNA isolation system. After the isolation, the yield of total RNA was determined with Nanodrop ND-1000, its purity was identified by the absorbance (OD_260_/OD_280_), and the integrity was assessed by 1% of agarose gel electrophoresis [35]. GoScript Reverse Transcription System was used to reverse transcribe cDNA following the manufacturer’s instructions, 20 μL of reaction system contained 2 μg of total RNA, and the reaction procedures were 42°C for 60 min and 70 °C for 15 min.

After the reverse transcription, the cDNA was used as template to amplify specific genes. The target gene product was amplified using GoTaq qPCR Master Mix, and Real-Time PCR was performed on Roche 480II Fast Real-Time PCR System (Applied Biosystems) with the PCR conditions (95°C for 5 min, and 45 cycles of amplification at 95 °C for 15 sec, 60 °C for 30 sec and 72°C for 30 sec). The housekeeper gene (glyceraldehyde-3-phosphate dehydrogenase, GAPDH) was used as the internal control to normalize the mRNA amounts of the target genes. Primers used in this study were as follows: GAPDH (forward, 5′-AGGTGGTCTCCTCTGACTTC-3′; reverse, 5′-TACCAGGAAATGAGCTTGAC-3′); TNF-α (forward, 5′- TCAACCTCCTCTCTGCCATC-3′; reverse, 5′- CCAAAGTAGACCTGCCCAGA-3′); IL-6 (forward, 5′-CACGGCCTTCCCTACTTCAC-3′; reverse, 5′-TGCAAGTGCATCATCGTTGT-3′); COX-2 (forward, 5′-GGGAGTCTGGAACATTGTGAA-3′; reverse, 5′-GCACGTTGATTGTAGGTGGACTGT-3′) [34, 36, 37].

### Statistical analysis

Three independent experiments were conducted, and data were expressed as the mean ± standard deviation (SD). The mRNA amounts of non-stimulated RAW264.7 cells (control) were compared with that of LPS-stimulated RAW264.7 cells (negative control) or experimental cells with STSP-I. Inhibition effects were determined by comparing the mRNA amounts of negative control with that of experimental group, and data were expressed as a percentage. The significance of differences between means was evaluated by one-way analysis of variance (ANOVA), and statistical significance was assigned at P < 0.05.

## Results and discussion

### Isolation, purification and physicochemical properties of STSP-I

**Fig 1:**
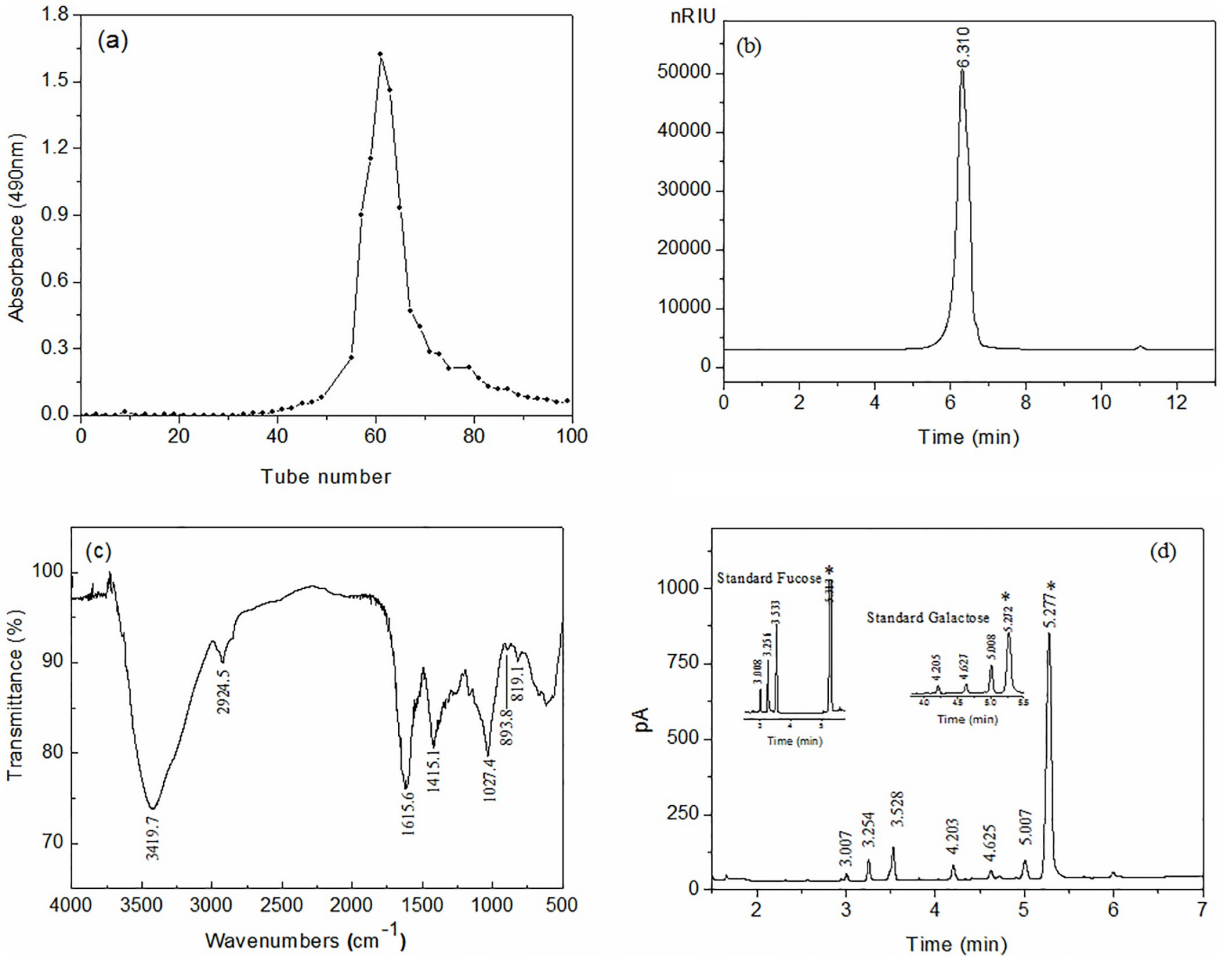
(a) Sugar curves of STSP-I from DEAE Sepharose CL-6B column chromatogram, (b) HPSEC chromatogram of STSP-I with a RID detector, (c) IR spectrum of STSP-I, and (d) GC chromatograms of TMCS derivative products from hydrolyzed STSP-I and standard samples. In the GC chromatograms, *asterisks* indicate the internal standard mannitol.

Crude products ST (41.67%, Dry to Dry weight ratio, D/D) were obtained with alkaline extraction. After purification by Sevage method and freeze-thawing cycles, ST gave crude polysaccharides STSP (18.67%, D/D), and STSP gave a polysaccharide fraction STSP-I (8.67%, D/D) from distilled water by purifying on the DEAE-Sepharose CL-6B column (Fig 1a).

The elute profile of STSP-I in HPSEC with a Sugar KS-804 column was given in Fig 1b, and STSP-I had uniform size with one singular narrow peak. Using HPSEC coupled with RID, a calibration curve (y = -4x+6.3) of standard dextrans was obtained, by which average molecular weights (Mw) of STSP-I was calculated to be 373 kDa. The purified fraction STSP-I was mainly composed of 98.91 ± 0.21% (w/w) carbohydrate and showed a negative response to the Bradford test. The result of the sulfate content test was negative, and the absence of absorption peaks at 1240 cm^-1^ (S = O) and 850 cm^-1^ (C-O-C) for sulfate esters indicated the STSP-I was a non-sulfated polysaccharide (Fig 1c) [38, 11]. The infrared spectrum of STSP-I displayed characteristic polysaccharide peaks at approximately 3419.7 cm^-1^ for hydroxyl groups, 2924.5 cm^-1^ and 1415.1 cm^-1^ for C–H band, 1615.6 cm^-1^ for C = O bond and 1027.4 cm^-1^ for C–O bond, and the absorption peaks at 893.8 cm^-1^ and 819.1 cm^-1^ confirmed the existence of *β*-glycosidic bonds and *α*-D-galactopyranose [38]. The monosaccharide composition of STSP-I was detected by GC analysis (Fig 1d), and STSP-I consisted mainly of fucose and galactose in a molar ratio of 1.2:1. Trimethylsilyl derivatives of monosaccharide obtained from aqueous solution tend to give several peaks, there is no overlap between the peaks of derivatives from fucose and galactose, and trimethylsilyl derivatives can be used for reliable qualitative and quantitative determination of these components in polysaccharide hydrolyzate by the retention times and a ratio of the corresponding peak areas [30].

### Structural characterization of STSP-I

In the study of periodate oxidation-Smith degradation, STSP-I showed 0.228 mmol of HIO_4_ uptake while it was oxidized after 7h, the oxidized-reduced products were used for Smith degradation, and the degraded polysaccharides were identified by GC (Table 1). The presence of fucose indicated that linkages of fucose in STSP-I were non-oxidized (1→3)-glycosidic linkages, the absent of galactose and the presence of erythritol suggested that linkages of galactose in STSP-I were oxidized (1→4)-glycosidic linkages or (1→4,6)-glycosidic linkages. There was no the sediment (S4), indicating the main chain was completely degraded in Smith degradation, and the results suggested further the non-oxidized 3-linked fucose and the oxidized 4-linked galactose were alternately arranged, namely alternating Fuc-Gal sequence. The conclusions were further supported by the detection of 3-linked fucose and 4-linked galactose in methylation–GC-MS and NMR.

**Table 1:** GC results from fractions of partial acid hydrolysis and smith degradation of STSP-I.

	Fractions	Sugar components and molar ratios			
		Fucose	Galactose	Glycerol	Erythritol
*Partial acid hydrolysis*	Precipitation	0.98	1		
	Out of sack	2.3	1		
	Supernatant in the sack	0.94	1		
*Smith degradation*	S1	+	-	-	+
	S2	-	-	-	+
	S3	+	-	-	-

-: Undetectable; +: Detectable.

The results of GC analysis after partial acid hydrolysis of STSP-I were showed in Table 1, fucose and galactose were identified in the fractions of precipitation and supernatant in the sack, and their molar ratios were close to the previous result of monosaccharide composition (Fuc:Gal = 1.2:1), indicating that fucose and galactose with the same molar number were composed of the backbone structure in STSP-I.

STSP-I was methylated, and methylated STSP-I was applied to the GC-MS analysis. Two components were obtained, namely 1,4,5-*O*-Ac_3_-2,3,6-*O*-Me_3_-D-galactiol and 1,3,5-*O*-Ac_3_-2,4-*O*-Me_2_-6-deoxy-L-galactiol with a molar ratio of 1.09:1, which indicated that galactose and fucose were 1,4-D-Gal*p* and 1,3-L-Fuc*p*, respectively. The above results were in consistent with the results of periodate oxidation-smith degradation. The branched fraction of STSP-I was not identified, and STSP-I was a non-branched polysaccharide, which was further confirmed by the NMR and TEM results.

**Fig 2:**
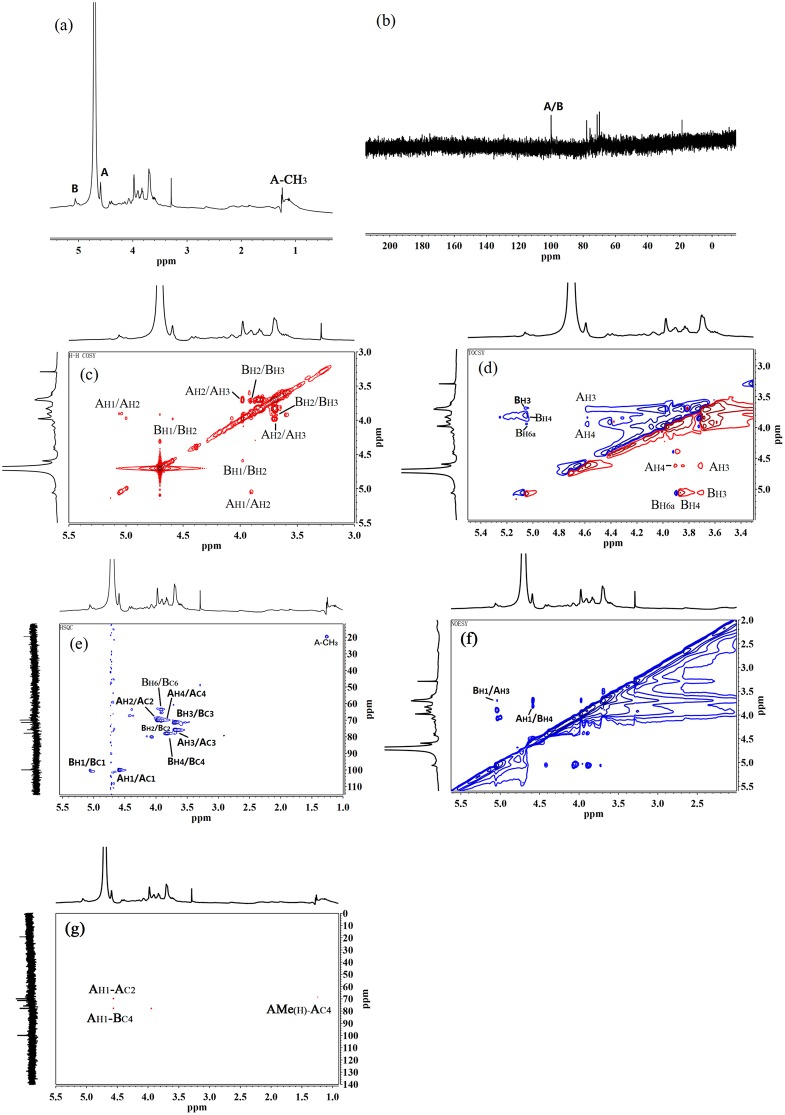
^1^H NMR (a), _13_C NMR (b), ^1^H-^1^H COSY (c), TOCSY (d), HSQC (e), NOESY (f) and HMBC (g) spectra of STSP-I. The anomeric protons were labeled A and B.

For the identification of sugar residues from STSP-I, NMR experiments were performed, including ^1^H (Fig 2a), _13_C (Fig 2b), ^1^H-^1^H COSY (Fig 2c), TOCSY (Fig 2d), HSQC (Fig 2e), NOESY (Fig 2f) and HMBC (Fig 2g) spectra. In the Fig 2a, two signals in anomeric regions of δ 4.59 ppm (s, *J*_H1-H2_ <3) and δ 5.06 ppm (s, *J*_H1-H2_ <3) with the molar ratio 0.98:1 were obtained, and they were assigned to residues A and B, respectively. The anomeric carbon signals appeared at δ 100 and δ 99.9 ppm in the _13_C NMR, and they were further confirmed to be corresponding C-1 chemical shifts of residues A and B in the Fig 2e. ^1^H assignments of residue A (H-2, H-3, H-4) and residue B (H-2, H-3, H-4, H-6a) were obtained through the ^1^H-^1^H COSY and TOCSY spectra, their carbon signals were identified in the Fig 2e, and the ^1^H and _13_C chemical shifts of residues A and B were listed in Table 2. In the Fig 2e, a cross peak with chemical shifts δ 1.21 and δ 18.6 ppm was also confirmed the methyl (–CH_3_) group derived from the residue A (C-6).

**Table 2:** ^1^H and ^13^C chemical shifts of the STSP-I.

Label	Glycosyl residues	chemical shifts (δ)						
			1	2	3	4	5	6
A	→3)-β-L-Fuc*p*-(1→	H C	4.59 100.0	3.98 69.9	3.70 75.8	3.93 70.1	- -	1.21 18.6
B	→4)-α-D-Gal*p*-(1→	H C	5.06 99.9	3.91 70.1	3.70 71.4	3.82 77.9	- -	3.90 63.2

-: Not obtained.

For the furanose configuration, the anomeric carbon appeared at δ 103–112 ppm, and the _13_C signal for the C-4 carbon of aldose or the C-5 carbon of ketose was obtained at δ 82–88 ppm. According to the _13_C NMR, there were no carbon signals at δ 82–88 ppm, and so residues A and B of STSP-I were in the pyranose form [39]. The anomeric carbon signal was seen at δ<100 ppm and its *J*_H1-H2_ were less than 3 Hz, which indicated that this residue was α-D or β-L configuration, and the anomeric carbon signal of δ>100 ppm and *J*_H1-H2_ of 6–8 Hz indicated the existence of β-D or α-L configuration [39]. The anomeric hydrogen signals of A and B in the polysaccharide STSP-I were single peaks (*J*_H1-H2_ < 3), both anomeric carbon signals were obtained at δ100 ppm, and the results gave evidence on α-D or β-L linked residues. According to the methylation-GC-MS, D-galactiol and 6-deoxy-L-galactiol were detected, and then the residues of STSP-I were α-D-galactose and β-L-fucose.

According to the ^13^C chemical shifts, the downfield shifts of A_C3_ (δ 75.8 ppm) and B_C4_ (δ 77.9 ppm) were obtained, and the residue A and B were identified as →3)-β-L-Fuc*p*-(1→ and →4)-α-D-Gal*p*-(1→, respectively.

Inter-residue connectivities were obtained in the NOESY spectrum (Fig 2f), the residue A had an NOE contact from H-1 to H-4 of the residue B, and the residue B had an NOE contact from H-1 to H-3 of the residue A. In the Fig 2g, long-range _13_C-^1^H correlations were obtained, and a cross-peak was observed between the H-1 (δ 4.59 ppm) of A and the C-4 (δ 77.9 ppm) of B. The results of NOESY and HMBC spectra indicated the alternating Fuc-Gal sequence in the polysaccharide STSP-I. The terminal fractions of STSP-I were not identified in the methylation-GC-MS and NMR analyses, probably because their contents were the least among the residues in linear STSP-I.

Based on the results of monosaccharide composition, periodate oxidation-Smith degradation, methylation-GC-MS and NMR, the following repeating unit was obtained.

$$\to [3)-\beta -\text{L}-\text{Fuc}p-(1\to 4)-\alpha -\text{D}-\text{Gal}p-{\left(1\right]}_{n}\to$$

### Chemical observation of STSP-I

**Fig 3:**
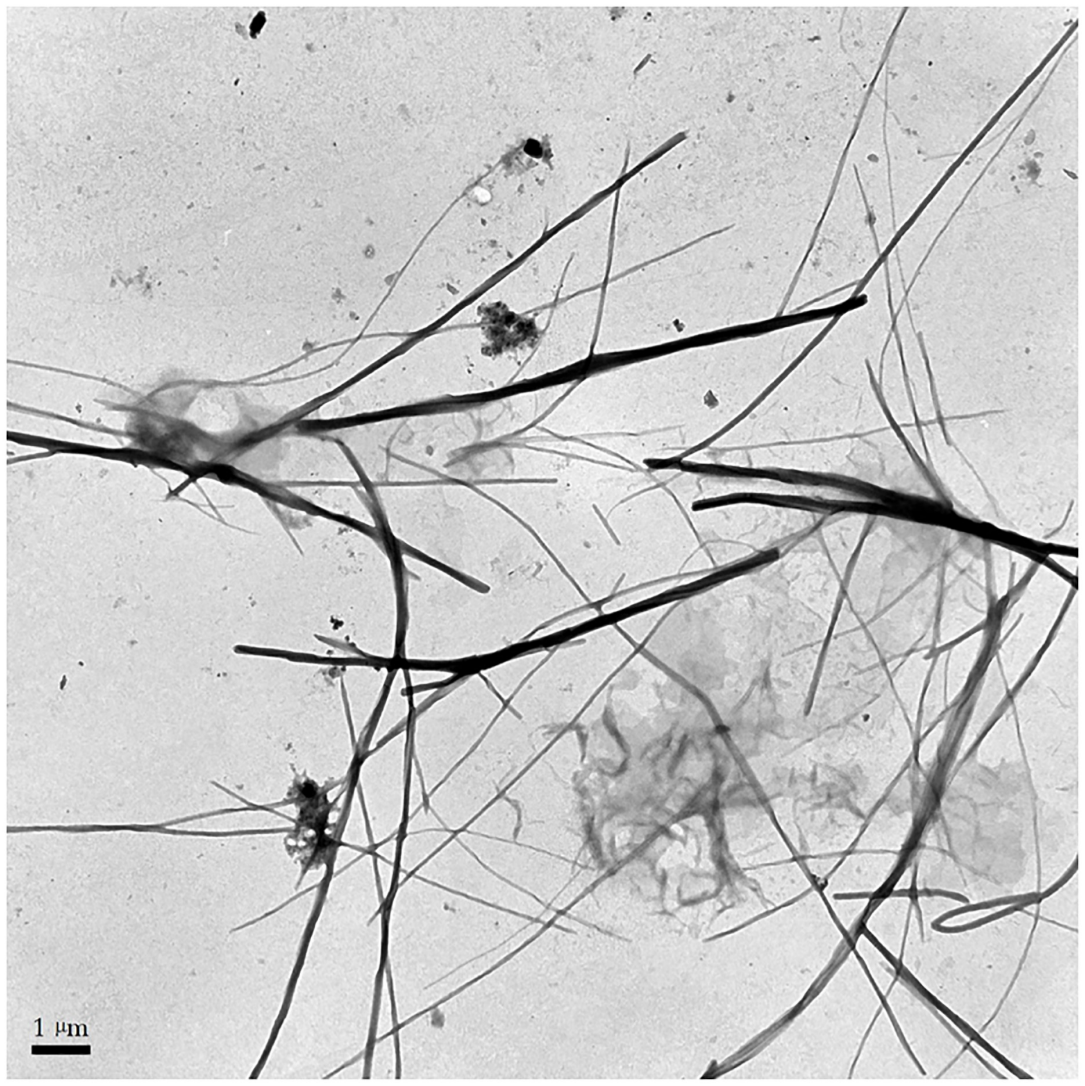
TEM image of STSP-I in sodium dodecyl sulfate (SDS) solution.

TEM was performed to illustrate the fine structure of macromolecules, and SDS was added to disperse the STSP-I [40]. Fig 3 showed a portion of the molecule STSP-I with extended conformations, and a linear chain with non-branched conformation was observed from the TEM image of STSP-I, which was in agreement with the results of NMR experiments. In addition, STSP-I molecules aggregated to form entangled chains under the experimental conditions, which contained more than two chains. It has been reported that entangled chains were beneficial for improving bioactivities of polysaccharides [41], which was further confirmed by antioxidant and anti-inflammation effects of STSP-I.

### Antioxidant and anti-inflammation activities of STSP-I

**Fig 4:**
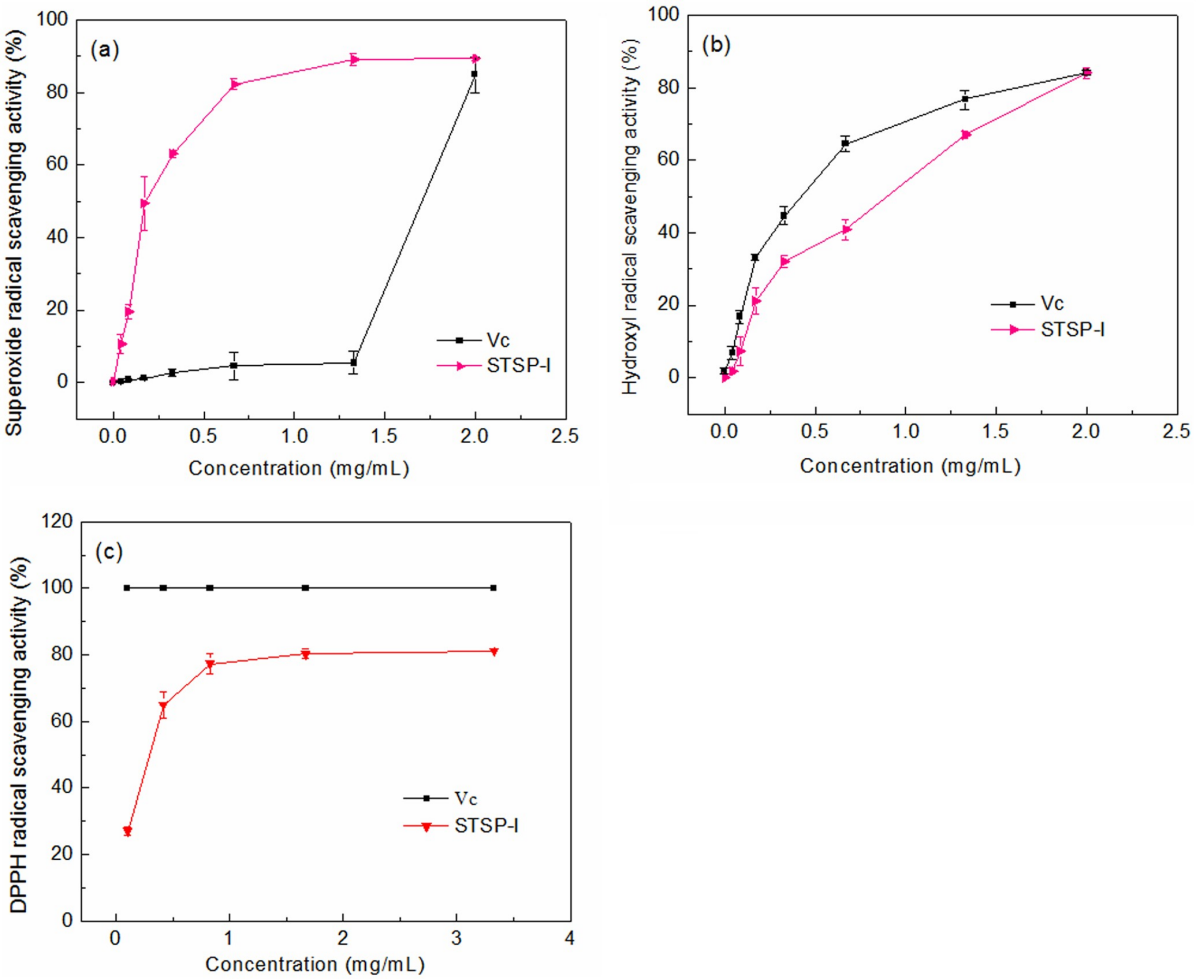
Antioxidant activities of STSP-I: (a) superoxide radical scavenging activity, (b) hydroxyl radical scavenging activity, and (c) DPPH radical scavenging activity. Each data *point* represents the mean ± SD (*n* = 3).

Scavenging assays for superoxide, hydroxyl and DPPH radicals were performed with the positive control Vc, and EC_50_ values were calculated for the identification of the antioxidant activities. The scavenging effects of STSP-I and Vc on the radicals were showed in Fig 4, and the scavenging activities increased with increasing concentrations. The EC_50_ values of Vc and STSP-I were 1.67 mg/mL and 0.22 mg/mL for superoxide radicals, 0.51 mg/mL and 0.88 mg/mL for hydroxyl radicals, 2.72 μg/mL and 260 μg/mL for DPPH radicals, respectively. The results of the EC_50_ values showed that STSP-I had a higher scavenging effect on superoxide radicals than Vc, and STSP-I also showed good scavenging effects on hydroxyl and DPPH radicals.

The cytotoxicity induced by STSP-I was investigated in RAW 264.7 mouse macrophage cells using the MTT assay, and STSP-I could not inhibit the proliferation of RAW 264.7 cells at the 75–150 μg/ml concentration ranges. In the next anti-inflammation experiments, the sample concentrations of STSP-I were used as 75 and 150 μg/ml, which had no effects on the growth of RAW 264.7 mouse macrophage cells.

**Fig 5:**
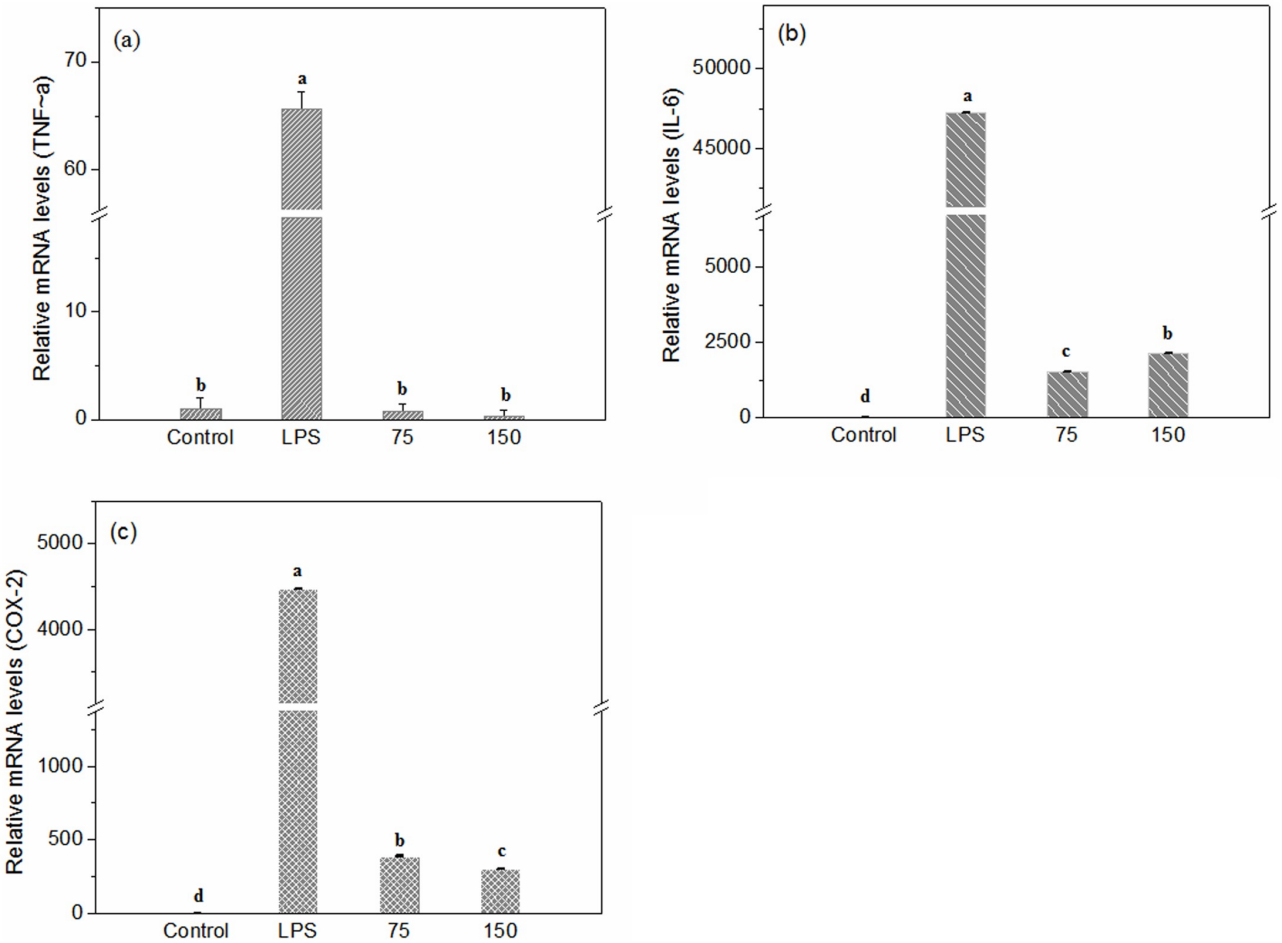
Effects of STSP-I on LPS-stimulated mRNA expressions of TNF-α (a), IL-6 (b) and COX-2 (c) in RAW264.7 mouse macrophage cells. 75 and 150 stand for the final treatment concentrations of samples at 75 μg/mL and 150 μg/mL followed by LPS inducement for 4h. Each data point represents the mean ± SD (*n* = 3). Different letters (a-d) indicate significant differences between groups (P<0.05).

To identify anti-inflammation bioactivities of STSP-I, the inhibitory effects on TNF-α, IL-6, and COX-2 mRNA expressions in LPS-stimulated RAW 264.7 mouse macrophage cells were evaluated. According to the absolute C_T_ values, the relative mRNA level was calculated by 2^-ΔΔCT^, and the results were showed in Fig 5. By the comparison with the control group, the group with adding 1 μg/mL of LPS significantly increased the level of TNF-α, IL-6 and COX-2 mRNA expression in RAW 264.7 cells (P<0.05), which indicated that the inflammatory model was successfully established. According to the comparison of LPS and sample groups, TNF-α, IL-6 and COX-2 mRNA expressions were reduced to 98.8%, 96.8% and 91.4% with the 75 μg/mL STSP-I, and 99.5, 95.5 and 93.2% of mRNA expressions were suppressed with 150 μg/mL STSP-I. STSP-I obviously reduced the LPS-induced TNF-α, IL-6 and COX-2 mRNA expressions (P<0.05), and the results suggest that STSP-I have good anti-inflammation activities.

## Conclusions

It was concluded that a polysaccharide STSP-I (8.67%, D/D) was obtained from the alkaline-extraction crude polysaccharide, and it was identified to be uniform size with the molecular weight of 373 kDa following the HPSEC analysis. The FTIR of STSP-I displayed the characteristic polysaccharide peaks. By using the GC analysis, STSP-I consisted mainly of fucose and galactose with the molar ratio of 1.2:1. According to the results of periodate oxidation-Smith degradation, partial hydrolysis, methylation-GC-MS and NMR, it was concluded that →4)-α-D-Gal*p*-(1→ and →3)- β-L-Fuc*p*-(1→ was composed of the main-chain structure of STSP-I, and STSP-I was a non-branched polysaccharide. TEM analysis revealed that STSP-I had linear microstructures with entangled chains, and the non-branched structure also was established. Due to the results of antioxidant and anti-inflammation activities, STSP-I with the EC_50_ values 1.67 mg/mL showed a higher scavenging activity for superoxide radicals than Vc, and TNF-α, IL-6 and COX-2 mRNA expressions were reduced to 99.5, 95.5 and 93.2% with 150 μg/mL STSP-I in LPS-stimulated RAW 264.7 mouse macrophage cells, which indicated that STSP-I showed notable anti-inflammation effects.
